# Effectiveness of methods for detaching orthodontic implants likely to fracture upon rotational torque – an animal study

**DOI:** 10.1002/cre2.20

**Published:** 2016-02-09

**Authors:** Susumu Nakagaki, Masahiro Iijima, Yoshitaka Yasuda, Keisuke Handa, Toshiyuki Koike, Takashi Saito, Itaru Mizoguchi

**Affiliations:** ^1^ Division of Orthodontics and Dentofacial Orthopedics, Department of Oral Growth and Development, School of Dentistry Health Sciences University of Hokkaido Ishikari‐Tobetsu Japan; ^2^ Yasuda Orthodontic Office Nishinomiya Japan; ^3^ Department of Restorative Dentistry Tohoku University Graduate School of Dentistry Sendai Japan; ^4^ Division of Clinical Cariology and Endodontology Department of Oral Rehabilitation, School of Dentistry, Health Sciences University of Hokkaido Ishikari‐Tobetsu Japan

**Keywords:** CBCT, fracture, miniscrews, piezosurgery, ultrasonic scaler

## Abstract

Orthodontic implants may fracture at the cortical bone level upon rotational torque. The impacted fragment can be detached by a range of methods, which are all more or less time‐consuming and injurious to the cortical bone. The aim of this study was to compare three different methods for detaching an orthodontic implant impacted in cortical bone. Health Sciences University of Hokkaido animal ethics committee approved the study protocol. Orthodontic titanium‐alloy (Ti‐6Al‐4 V) implants were placed bilaterally on the buccal side of the mandible of beagle dogs. Subsequently, the implants were detached using either a low‐speed handpiece with a round bur, alternatively by use of a low‐power or a high‐power ultrasonic instrument. In the first experiment, 56 orthodontic implants were placed into the dissected mandible from 7 animals. The methods for detachment were compared with respect to time interval, as well as associated undesirable bone loss as appraised by use of cone‐beam computed tomography. In experiment two, 2x2 implants were placed bilaterally in the mandible of 8 animals and subsequently detached by manual rotational torque, and the described three methods for detachment. The implant socket was investigated histologically as a function of removal method immediately after removal, and after 1, 3 and 8 weeks and contrasted with the healing of the socket of the implant that was detached by manual rotational torque. Statistical significance was appraised by the use of non‐parametric Kruskal‐Wallis one‐way analysis of variance. The method using the low‐power ultrasonic required significantly longer removal time versus the two other methods, i.e. high‐power ultrasonic and low‐speed handpiece with a round bur (p < 0.02). The amount of undesirable bone loss was substantially larger with low‐speed handpiece with a round bur compared to the two ultrasonic methods (p < 0.05). Bone formation after 3 weeks of healing was more complete following the use of low or high‐power ultrasonic instrument in comparison with a low‐speed handpiece rotary instrument method. Orthodontic implants likely to fracture upon rotational torque or impacted fractured fragments should be detached preferably with an ultrasonic instrument, because of less associated bone loss and more rapid bone healing compared to the use of a low‐speed handpiece rotary instrument.

## Introduction

Miniscrew implants were introduced in clinical orthodontics by Kanomi (1997). They have gained popularity because of their small size that allows for multiple placement sites in the oral cavity, a marginal discomfort for the patient, an easy surgical procedure, and relatively low costs (Kuroda et al., 2007; Miyawaki et al., 2003; Park et al., 2006). However, the success rate of orthodontic implants is less than for dental implants, reported to range between 6% and 30% (Deguchi et al., 2009; Kuroda et al., 2007; Miyawaki et al., 2003; Park et al., 2006; Schätzle et al., 2009). The relatively lower success rate of orthodontic implants are associated with trauma to the periodontal ligament or the dental root, nerve involvement, and nasal and maxillary sinus perforation (Kravitz and Kusnoto, 2007). Decreasing the diameter of the implant may theoretically reduce the risk of trauma to the periodontal ligament and the dental root, but will concurrently lower the fracture resistance of the orthodontic implant (Barros et al., 2011; Iijima et al., 2008; Wilmes et al., 2011).

Recent clinical studies have reported that approximately 2 to 7% of orthodontic implants fracture during their placement or removal (Büchter et al., 2005; Chen et al., 2006; Park et al., 2006). Another recent survey reported that 9% of orthodontists have experienced fracturing of an orthodontic implant (Hyde et al., 2010). An orthodontic implant that fractures during placement or removal can create a considerable problem if the embedded fragment remain firmly impacted in the bone. Detaching an embedded fragment may require a surgical exposure of the site with a full‐thickness flap and subsequent removal of bone around the implant (Smith et al., 2015). The digging procedure using a low‐speed handpiece with a bur may cause not only mechanical trauma, but also a risk of heat‐induced bone necrosis in cortical bone (Canullo et al., 2014; Erikson et al., 1982). However, little information is available in the literature on methods for removing orthodontic implants likely to fracture upon rotational torque or actually fractured fragments impacted into cortical bone.

High‐power piezoelectric ultrasonic instruments with a cutting ability in bone have been used for various maxillofacial surgical operations (Eggers et al., 2004), such as dental implant site preparation (Canullo et al., 2014), and removal of impacted third molars (Sortino et al., 2008). The use of ultrasonic instruments may be also an option for detaching orthodontic implants or implant fragments embedded in bone. The current study aimed to compare the outcomes following the use of two ultrasonic instrument methods versus the prevailing method of using a low‐speed handpiece with a rotating instrument.

## Materials and Methods

The study protocol was approved by the Animal Ethics Committee of The Health Sciences University of Hokkaido (No.063).

All orthodontic implants in this study were made from a titanium alloy (Ti–6Al–4 V) and were 7 mm long with a 1.3‐mm tip diameter, 1.4‐mm neck side diameter (AbsoAnchor SH‐1413‐07, Dentos, Daegu, Korea).

### Experiment one, removal time and bone loss

Seven healthy male beagle dogs (age, 10–15 months; weight, ~10 kg) were selected. The animals were anesthetized and sacrificed with thiopental sodium (Ravonal, Mitsubishi Tanabe Pharma; 150 mg per kg, i.v.), and the mandible were dissected free. Fifty‐six orthodontic implants (four implants on side of the mandible) were manually placed in the interradicular and interdental areas of the first and second mandibular molars using a hand‐driver without drilling a pilot hole, and then the implants were detached with one of the following methods by the same operator.

- Application of rotational torque manually. The size of the socket of the orthodontic implant removed with a hand‐driver was compared to the socket sizes generated by applying the three different methods for detaching the implant.
- A low‐power ultrasonic instrument (Piezon Master 700, Shofu, Kyoto, Japan), generally known as an ultrasonic scaler, with maximum power of 8 W (watt) and 24–32‐kHz piezoelectric frequencies, was applied with light pressure around the implant periphery.
- A high‐power ultrasonic instrument (Piezon Master Surgery, Shofu) with maximum power 25 W (watt) and 24–32‐kHz piezoelectric frequencies, with bone cutting ability for ultrasonic osteotomy (piezosurgery) in dental implantology (Eggers et al., 2004; Parmar et al., 2011; Canullo et al., 2014) was applied with light pressure around the implant periphery.
- A low‐speed handpiece with a rotating instrument consisting of a round steel bur; the bone surrounding the orthodontic implant was removed using light pressure and a speed of 2,000 r.p.m.

**Figure 1:**
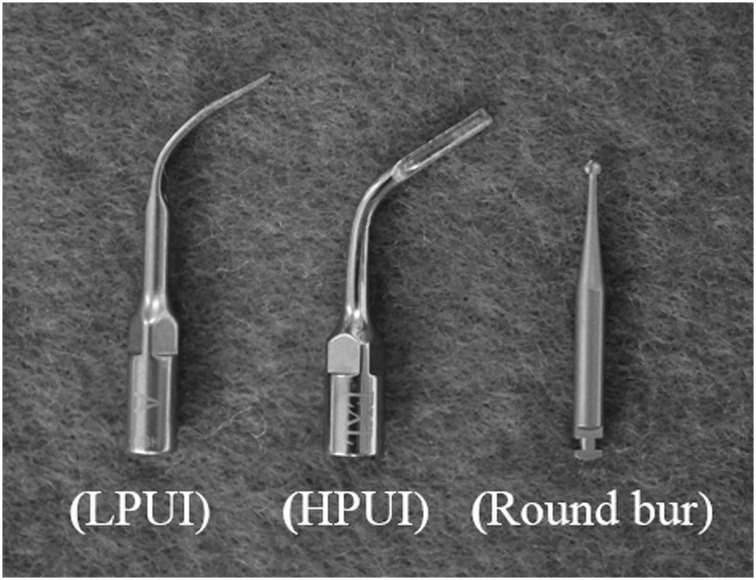
Morphological features of the tips of the ultrasonic instruments and the low‐speed handpiece rotary instrument, consisting of a round steel bur.

**Figure 2:**
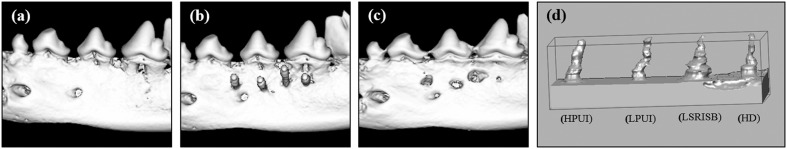
Three‐dimensional images obtained by CBCT at (a) before placing the orthodontic implant, (b) after placing the orthodontic implant and (c) after removing the orthodontic implant. (d), reversal 3D images of undesirable bone associated with detaching the impacted orthodontic implant. (HD = Hand driver, LPUI = Low‐power ultrasonic instrument, HPUI = High‐power ultrasonic instrument, LSRISB = Low speed rotating instrument steel bur).

The ultrasonic and rotating instruments are shown in Figure 1. During all procedures except for the method using the hand‐driver, the ultrasonic and rotating instruments were cooled with physiologic sterile saline solution at 4 °C. Once the implant became mobile, it was removed from the bone by the operator's fingers. The time needed to detach the orthodontic implants was recorded and compared. The dissected mandibles scanned in by cone‐beam computed tomography (CBCT) before and after placing the orthodontic implants, and after having detached the implants. The CBCT had a tube voltage of 85 kV and tube current 6 μA that delivers approximately 0.1 mm in voxel size (AUGE X ZIO CM, Asahi Roentogen, Kyoto, Japan). The data recorded were transformed into Digital Imaging and Communications in Medicine (DICOM) format and reconstructed to a three‐dimensional (3D) image using imaging software (V‐works 4.0, CyberMed). The 3D images obtained by CBCT at each step were also used to generate reversal images of undesirable bone loss for each removal method (Fig. 2). The amounts of bone loss (surface area and volume) were determined by the applying a reverse engineering software (RapidForm 2006, Inus Technology)

### Experiment two, bone‐healing following implant removal

The bone‐healing process following the implant removal was investigated histologically. Eight healthy male beagle dogs (age, 10–15 months; weight, ~10 kg) were selected. The animals were treated with ketamine hydrochloride (Ketalar, Daiichi Sankyo Espha, Tokyo, Japan; 5 mg per kg, i.m.) for analgesia and thiopental sodium (Ravonal, Mitsubishi Tanabe Pharma, Osaka, Japan; 50 mg per kg, i.v.) for anesthesia. Diazepam (Horizon, Astellas Pharma, Tokyo, Japan; 2 mg per kg) and atropine sulfate (Mitsubishi Tanabe Pharma; 0.1 mg per kg) were given intramuscularly for pre‐anesthesia. Thirty‐two orthodontic implants (two implants on each side of the mandible), 1.3 mm in tip diameter, 1.4 mm in neck side diameter, and 7 mm in length, were manually placed in the mesiodistal areas of the second molar using a hand‐driver without drilling a pilot hole. The mobility of the orthodontic implants was checked with dental tweezers immediately after placement. The orthodontic implants were subsequently removed with the hand‐driver or by the three methods for detaching implants as described above. Four periods of healing were compared, i.e., 0 week, 1 week, 3 weeks and 8 weeks (n = 2 animals for each). At each period, the animals were anesthetized and sacrificed with thiopental sodium (Ravonal, Mitsubishi Tanabe Pharma; 150 mg per kg, i.v.), and the mandible was dissected free. The mandible specimens were sectioned to an approximate size of 10 × 10 × 10 mm^3^ using a slow‐speed water‐cooled diamond saw (Isomet 11–1280, Buehler). The specimens were fixed in 10% formaldehyde neutral buffer solution for 2 weeks at room temperature and then encapsulated in epoxy resin (Epofix, Struers, Copenhagen, Denmark). The specimens were sectioned in the longitudinal plane with a micro‐cutting machine (BS‐300, Exakt Advanced Technologies, Hamburg, Germany). The thick slices were ground and polished to a thickness of ~30 µm with a microgrinding machine (MG‐4000, Exakt Advanced Technologies). The slices were subsequently stained with hematoxylin‐eosin and observed under a light microscope at x100 magnification to assess the healing state.

### Statistical analysis

Statistical analysis was performed using PASW Statistics (version 18.0 J for Windows, IBM, Armonk, NY, USA). The time to detach the implant and the bone loss (surface areas and volumes) were not normally distributed according to the Levene test. Hence, Kruskal–Wallis one‐way analysis of variance tests were applied to determine whether significant differences existed among the study groups.

## Results

**Figure 3:**
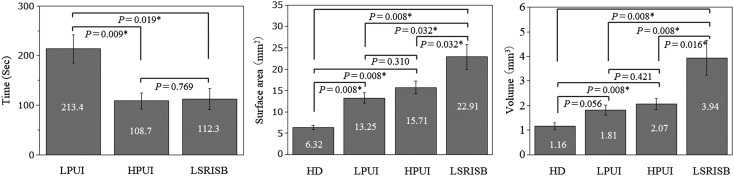
Comparison of removal times (seconds), surface areas (mm^2^) and volumes (mm^3^) of bone loss for each of the methods for detaching the orthodontic implant. *P‐values according to Kruskal–Wallis one‐way analysis of variance tests. (HD = Hand driver, LPUI = Low‐power ultrasonic instrument, HPUI = High‐power ultrasonic instrument, LSRISB = Low speed rotating instrument steel bur).

The times required to detach the orthodontic implants and the amounts of undesirable bone loss (surface areas and volumes) are shown in Figure 3. The average time for detaching the implant was significantly longer when a low‐power ultrasonic instrument was used (213 sec.), compared with the two other methods (109 sec. for high‐power ultrasonic instrument, and 112 sec. for rotating instrument) (p < 0.05). The bone loss were fairly similar for the two ultrasonic instrument methods and significantly less than for rotating instrument method, measured by surface area as well as by volume (p < 0.05).

**Figure 4:**
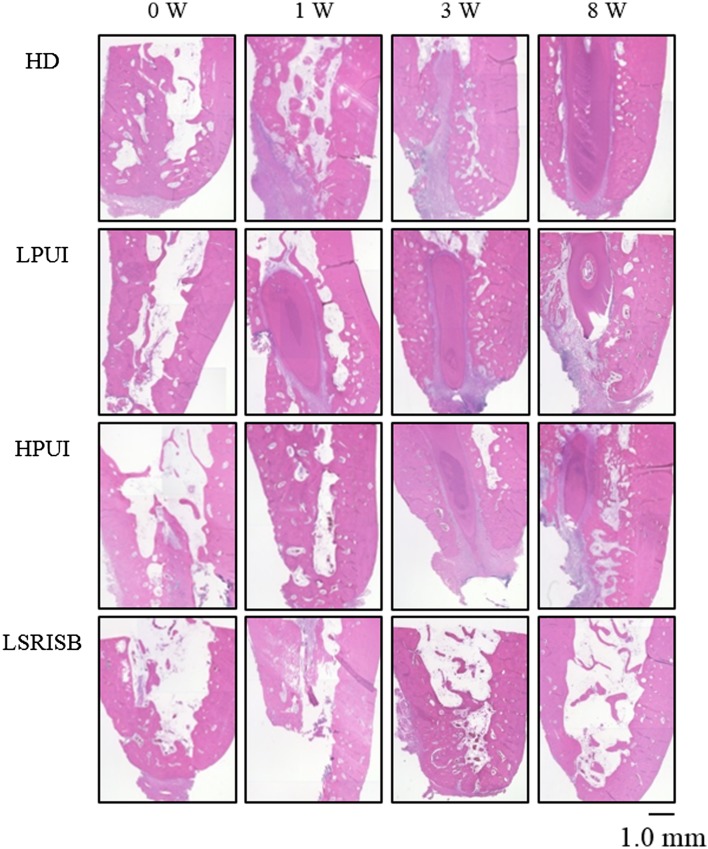
Representative optical micrographs (original magnification, x100) at each healing period (0, 1 week, 3 weeks, 8 weeks). (HD = Hand driver, LPUI = Low‐power ultrasonic instrument, HPUI = High‐power ultrasonic instrument, LSRISB = Low speed rotating instrument steel bur).

Figure 4 shows representative optical micrographs obtained at each healing period (0, 1, 3, 8 weeks). The specimen from which the implant was detached using a hand‐driver had an empty cavity (area of bone loss) after removal of the orthodontic implant. The cavity was filled with granulation tissue after 1 week of healing, which was replaced with new bone after 3 weeks of healing. Eventually, maturity of the new bone was observed in a typical bone healing process. Although the specimens on which the ultrasonic methods were used showed slower healing at 1 week compared with the hand‐driver specimen, similar bone formation, with histological features similar to those of the hand‐driver specimen, was observed at 3 weeks and 8 weeks. The specimen removed with the rotating instrument method showed a comparatively large cavity in the bone, which was still obvious due to delayed bone formation 8 weeks after the implant removal.

## Discussion

The present study confirmed that methods using ultrasonic instruments are preferable for removing an implant prone to fracture or an actual fractured fragment embedded in bone because of less bone loss compared with a method using a low‐speed handpiece with a round bur.

Low‐power ultrasonic instruments have long been used in dentistry for removing plaque and calculus. Ultrasonic instrument with higher power was developed in the late eighties and is based on a modulated ultrasonic frequency that permits the cutting of hard tissue (Stübinger et al., 2005). The high‐power ultrasonic instrument can cut bone mechanically without damaging the surrounding soft tissues and these instruments have therefore been extensively employed for various surgical operations (Eggers et al., 2004). In the present study, the high‐power piezoelectric ultrasonic instrument showed significantly quicker removal of orthodontic implants from bone, as compared with a conventional ultrasonic instrument, which was comparable to the method using a conventional low‐speed handpiece with a round bur. It is also possible to use a low‐power ultrasonic instrument to detach a orthodontic implant fragment from bone, although a longer time will be required. Ultrasonic instrument methods enable a more rapid healing because of less heat‐induced bone necrosis (Canullo et al., 2014; Erikson et al., 1982), which was corroborated by the histological findings in the present study. The specimens on which ultrasonic instruments were used showed favorable formation of new bone after 3 weeks of healing, and eventually their maturity was similar to the specimen removed with a hand‐driver (Fig. 4).

Commercially available orthodontic implants with various shapes may show a wide range of torques at fracture (Iijima et al., 2008; Smith et al., 2015). Increasing the diameter can result in increasing fracture resistance of orthodontic implants (Barros et al., 2011; Iijima et al., 2008; Wilmes et al., 2011), although using a orthodontic implant with greater diameter increases the risk of trauma to the periodontal ligament and the dental root. On the other hand, a self‐drilling orthodontic implant has some advantages, such as superior primary stability and reduced risks to the periodontal ligament and the dental root, and tends to generate higher insertion torques than self‐tapping orthodontic implants (Chen et al., 2008; Suzuki and Suzuki, 2011). Clinicians must understand various factors that influence the values of insertion and removal torques, such as the density and quality of bone, thickness of cortical bone, and design and size of the implants. If clinical insertion torque values are expected to be close to the fracture torque, hand‐operated and battery‐operated torque‐limiting devices should be used to inhibit orthodontic implant fracture (Pauls et al., 2013). In addition, a pilot hole is recommended in dense regions and thick cortical bone before using self‐drilling orthodontic implants.

The bone‐to‐implant apposition may for some patients becomes so extensive that a rotational torqueing during removal will fracture the implant. Also, fluoride‐containing products, such as toothpaste and mouthrinse, are commonly used in dentistry to prevent dental caries, although fluoride ions in the product may combine with hydrogen ions to form hydrogen fluoride (HF), which attacks the protective surface oxide of titanium alloys used for implants and leads to brittle fracture of titanium products (Rodrigues et al., 2009). Recent studies investigated retrieved orthodontic implants (Eliades et al., 2009; Iijima et al., 2015) and reported that minimal degradation of the bulk mechanical properties of orthodontic implants was observed after clinical use, although precipitation of bone‐like structures, formation of a carbonaceous contamination layer, and hydrogen absorption were observed on the surfaces of the implants.

## Conclusion

Within the limitation of this study, we concluded that methods using ultrasonic instruments are preferable for removing orthodontic implants likely to fracture upon rotational torque or impacted fractured fragments because of less bone loss and more rapid bone healing compared with a method using a low‐speed handpiece rotary instrument.

## Conflict of Interest

All authors affirm that they have no commercial association that might pose a conflict of interest for the subject or the materials discussed in this manuscript.
